# Value-based Reimbursement as a Mechanism to Achieve Social and Financial Impact in the Healthcare System

**DOI:** 10.36469/001c.89151

**Published:** 2023-10-31

**Authors:** Ana Paula Beck de Silva Etges, Harry H. Liu, Porter Jones, Carisi A. Polanczyk

**Affiliations:** 1 Avant-garde Health, Boston, Massachusetts; 2 National Institute of Science and Technology for Health Technology Assessment, Porto Alegre, Brazil; 3 Graduate Program in Epidemiology Universidade Federal do Rio Grande do Sul School of Medicine, Porto Alegre, Brazil

**Keywords:** value-based reimbursement, value-based health care, payment reform, health policy

## Abstract

**Background:** Value-based reimbursement strategies have been considered in the continuous search for establishing a sustainable healthcare system. For models that have been already implemented, success is demonstrated according to specific details of the patients’ consumption profile based on their clinical condition and the risk balance among all the stakeholders. From fee-for-service to value-based bundled payment strategies, the manner in which accurate patient-level cost and outcome information are used varies, resulting in different risk agreements between stakeholders. A thorough understanding of value-based reimbursement agreements that views such agreements as a mechanism for risk management is critical to the task of ensuring that the healthcare system generates social impacts while ensuring financial sustainability. This perspective article focuses on a critical analysis of the impact of value-based reimbursement strategies on the healthcare system from a social and financial perspective.

**Methods:** A critical analysis of the literature about value-based reimbursement was used to identify how these strategies impact healthcare systems. The literature analysis was followed by the conceptual description of value-based reimbursement agreements as mechanisms for achieving social and financial impacts on the healthcare system.

**Results:** There is no single successful path toward payment reform. Payment reform is used as a strategy to re-engineer the way in which the system is organized to provide care to patients, and its successful implementation leads to cultural, social, and financial changes. Stakeholders have reached consensus regarding the claim that the use of value reimbursement strategies and business models could increase efficiency and generate social impact by reducing healthcare inequity and improving population health. However, the successful implementation of such new strategies involves financial and social risks that require better management by all the stakeholders. The use of cutting-edge technologies are essential advances to manage these risks and must be paired with strong leadership focusing on the directive to improve population health and, consequently, value.

**Conclusion:** Payment reform is used as a mechanism to re-engineer how the system is organized to deliver care to patients, and its successful implementation is expected to result in social and financial modifications to the healthcare system.

## BACKGROUND

The most commonly used payment system in healthcare worldwide is the fee-for-service model, which reimburses providers for the patient care they deliver without considering any metrics related to outcomes, which might result in a lack of equity and accountability in the care process. The non-consideration of outcomes in determining payments has motivated the creation of redesigned approaches to healthcare reimbursement, including the concept of value, which means delivering better outcomes without increasing costs.^1^ Value-based reimbursement strategies are innovative solutions that can be considered by healthcare policymakers who desire to establish a more sustainable healthcare system, and its dissemination motivated the creation by the ISPOR in 2022 of the Value-Based Healthcare Implementation Special Task Force. Evidence of the successful implementation of value reimbursement strategies is starting to emerge. For models that have already been put into practice, success is demonstrated according to specific details of the profile of patients’ consumption, due to their clinical condition, and the risk balance between all the stakeholders.^2^ From fee-for-service to the most recent bundled-payment strategies based on value, the manner in which accurate patient-level cost and outcome information are used varies, resulting in different risk agreements between stakeholders. By understanding the scope of this challenge, based on the care system perspective, no unique payment model has emerged and been followed as the gold standard strategy for reimbursing healthcare providers for services delivered to the patient population.

The search for continuous solutions to the healthcare crisis and improvement in the delivery of patient-focused health outcomes, a task that lies at the center of how organizations are managed, entails the need to prioritize patients and reimburse the system in light of patient outcomes.^3^ When using new reimbursement models to facilitate this patient-centered care process, the financial and social risks faced by all stakeholders should be considered. This situation of risk balancing was identified by Prof. Michael Porter as one of the main reasons why strategies such as bundled payments have the potential to attenuate the healthcare crisis.^1^ Once patients’ perceptions and outcomes that matter to them are used to establish reimbursement, all stakeholders (eg, pharmaceutical companies, providers, insurers, and government payers) are financially and socially motivated to deliver better outcomes. This approach serves as an additional and important incentive for stakeholders to achieve the goal that should be the primary objective of a company operating in the healthcare business: to generate sustainable social impact, which means increasing population health and equity while ensuring financial accountability.

Value-based reimbursement strategies must account for patient-level heterogeneity and risk stratification to be successful. This has been recently evidenced in a systematic review that evaluated the impact of innovative reimbursement strategies on the healthcare system.^2^ They demonstrated that although value-based reimbursement agreements have been recognized as strategies used to reduce waste in health care, in the real world, such agreements have led to an increase in total Medicare spending after the distribution of financial bonuses.^4^ To manage the financial risk of value-based reimbursement agreements more effectively, it is critical to consider accurate patient-level cost information when adjusting fees and bonuses before establihing new payment strategies.^5^

The achievement of outcome and cost measurement capabilities at the individual case level while ensuring compliance and agility requires innovative cutting-edge technologies.^6^ Those technologies have the capability to collect and evaluate data for any clinical condition or care cycle and to provide data that can be used to identify the most effective reimbursement strategies. Only by measuring the resource consumption more effectively at the individual case level is it possible to identify the reimbursement model that best fits each clinical condition.^7^ It is beneficial to leverage technologies that rely on the gold-standard cost accounting method in the context of value-based healthcare studies, that is, time-driven activity-based costing.^8^ Previous studies have demonstrated the contributions of time-driven activity-based costing to measuring, at the level of the individual or the clinical condition, the corresponding variability in resource consumption; consequently, this approach is a powerful instrument that can be used to provide accurate information to improve value-based reimbursement strategies.^9,10^

Considering the value-based reimbursement strategies that have been implemented, have we truly balanced risks among stakeholders and generated social impact? Have we improved healthcare equity and population health without increasing costs? A thorough understanding of value-based reimbursement agreements, including accurate cost and outcome information, that views such agreements as a mechanism for risk management is critical to the task of ensuring that the healthcare system generates social impacts while ensuring financial sustainability.

**Highlights**

- The impact of value-based reimbursement strategies implemented in the last decade are beginning to be published, identifying the obstacles that are limiting its extension into the healthcare system.
- The use of cutting-edge technologies to accurately measure cost and outcome information is required for administrators who work to promote a more sustainable healthcare system. However, these technologies must be paired with strong leadership focusing on the directive to improve population health and, consequently, value.
- Value-based reimbursement requires balancing risks among all stakeholders to achieve social impact, increase health equity, and improve population health without increasing costs.
- Establishing a healthcare system that delivers better outcomes and improves population health without increasing costs entails the generation of sustainable social impact, which means increasing population health and equity while ensuring financial accountability.

## Historical Aspects and Risk Balancing Regarding Healthcare Reimbursement Strategies

From 1980 to 2010, several value-based reimbursement strategies were considered, tested, and implemented, such as the Diagnosis Related Groups (DRG), which take into account patient complexity and case mix in the reimbursement process but do not account for outcomes or the quality of services delivered. This strategy remains a volume-based system that fuels waste.^11^ In recent decades, value-based reimbursement strategies have been introduced as alternatives that can be used to align the payer, the provider, and the patient.^12^ These strategies offer incentives to providers who deliver better patient outcomes and experiences without increasing costs. Two main approaches and their adaptations emerged: capitation, which was originally created as a population-based payment model, and bundled payments.

The capitation payment model provides a prespecified and fixed amount of money to providers who deliver complete care services to a population over a defined period of time, adjusted for patient case mix and the quality of care.^13^ By establishing a fixed price per patient as reimbursement for the complete care cycle, providers face the risk of losing money but are also motivated to improve efficiency and reduce waste.^14^ The healthcare provider assumes the financial risk. Due to the amount of financial risk taken by providers, they are incentivized to reduce or withhold necessary services. Capitation has frequently been criticized due to this adverse incentive to withhold care; in parallel, the strategy of bundled payments started to emerge.

In a bundled payment, providers are paid for the complete cycle of care for a specific clinical condition and may receive an incentive based on the outcomes achieved.^15^ A bundle includes all the services rendered from diagnosis to discharge throughout the episode of care, including all procedures, medications, and exams; it also often includes post-acute care.^16^ The aim of the bundled payment model is to prioritize the quality of the service delivered, decrease costs, and motivate all stakeholders to provide better patient care, especially in models that include a bonus.^1^ This bonus, in general, is paid at a professional level; this approach is expected to be sustainable because of the cost savings achieved in successful treatments. For example, when the patient’s length of stay in a case of arthroplasty surgery is decreased, it is possible to pay a prefixed bonus to the clinicians.^17^ This model distributes risk among payers, providers, and clinicians, thereby motivating them to exhibit competitiveness to elicit better results from clinical teams and centers.^18^ Because of these characteristics, this approach has been recognized as the best reimbursement strategy in value-based health care.^1^ Bundled payments were widely adopted after the passage of the Patient Protection and Affordable Care Act in the United States, which inaugurated the Bundled Payments for Care Improvement (BPCI) Initiative in 2013. This initiative included four models^17,19^:

- **Model 1** focused on acute care inpatient hospitalization with a standard discount to the usual Medicare hospital payment.
- **Model 2** focused on inpatient hospital services, physician services, and post-acute care services during a specific episode of care, including hospital readmissions.
- **Model 3** focused on post-acute care services during a specific episode of care, beginning after discharge from inpatient hospitalization.
- **Model 4** focused on all inpatient and physician services during the initial hospital stay and subsequent hospital readmissions but did not include post-acute care services. In this model, the payment was prospective at the start of an episode of care.^19^

BPCI was first introduced nationally as an innovative reimbursement method. Ten years from its initial implementation, published data have shown its impact in terms of reducing waste and inequity. For example, several studies on total joint arthroplasty surgeries have reported cost savings and improved outcomes resulting from reductions in hospital length of stay, hospital readmission rates, and post-acute care.^20^ A recent systematic review demonstrated that in the context of BPCI clinical episodes, total joint arthroplasty surgeries achieved the most significant results in terms of cost savings while maintaining or improving clinical outcomes.^2^ On the other side, the impact of bundled agreements on medical conditions such as congestive heart failure, pneumonia, chronic obstructive pulmonary disease, and acute myocardial infarction was not associated with significant changes in Medicare payments, emergency department use, length of stay, and hospital readmission. These findings suggest the value of designing new strategies and partnerships, or additional incentives, that starts from the beginning of care cycles in primary care.^21^

Primary care has become a key component of the country’s value-based reimbursement strategies. The Centers for Medicare & Medicaid Services launched the Comprehensive Primary Care Initiative, which included voluntary performance-based and risk-sharing payment agreements for primary care services.^22^ According to this model, advanced primary care practices receive a fee for the first consultation, a monthly population-based payment, and a quarterly performance-based payment that can increase revenue by 50% or decrease revenue by 10%. The performance evaluation is based on outcome measures, such as blood pressure and diabetes control, cancer screening, individual care planning, and patient experience. This program represents an important step in transforming the healthcare system to focus on value, but its implementation requires cost and outcome measurement capacity over the full episode of care for each individual and strategic modifications to the agreements between payers and providers.^23^

One example of systemic and strategic modification is in the field of cardiology. With the aim of reducing the burden of the leading cause of hospitalizations and readmission in the United States, the American Heart Association suggested the implementation of a value-based model with a longitudinal focus on heart failure management and prevention.^24^ The main barriers to the implementation of this model are its requirement of disruptive changes in organizational culture, professional behavior, and agreements among stakeholders. For example, the principles underlying value-based agreements in cases of heart failure include the search for more equitable and inclusive care for the entire population, which includes providing access to specialized care. However, the achievement of this goal is feasible only if coordination between primary and specialized care is improved, which would require significant changes in the way in which the system is organized.^24^

The sustainability of the BPCI agreements after the inclusion of bonus conditions has been associated with essential elements that must be managed by stakeholders. The first available evaluations have suggested the importance of accurately considering costs and the variability associated with the way in which patients interact with the healthcare system regarding the characteristics of each disease prior to the definition of the agreements to mitigate financial and social risks to the system.^5^ Regarding its impact on equity and population health, although the objective of these innovative solutions to the challenge of generating social impact by increasing value in the healthcare system seems to be clear, its successful implementation is dependent on an accurate measurement process for outcomes and costs, which must be followed by radical cultural and organizational changes in which providers, payers and patients must all participate.^25^

## Value-Based Reimbursement Strategies as a Mechanism to Achieve Social Impact While Ensuring Financial Sustainability

We suggest that achieving a healthcare system that delivers better outcomes and improves population health without increasing costs entails the generation of sustainable social impact (ie, increasing population health and equity while ensuring financial accountability). Stakeholders have reached consensus regarding the claim that the use of value reimbursement strategies and business models could increase efficiency and generate social impact by reducing healthcare inequity and improving population health. However, the successful implementation of such new strategies involves financial and social risks that require better management during the planning phase. When healthcare leaders representing all stakeholders do not address these organizational and financial risks with the aim of strategically aligning incentives with patient outcomes and generating a positive social impact, value-based reimbursement models tend to fail. Using real-world cost and outcome data to mitigate these risks and support decisions is key to the creation of transparency and trust and the promotion of engagement (among stakeholders) with the objective of increasing value in health care.

There is no single successful path toward payment reform. Payment reform is used as a strategy to re-engineer the way in which the system is organized to provide care to patients, and its successful implementation leads to cultural, social and financial changes to the healthcare system. The goal of this process is to design and successfully implement an evidence-based system that is equitable to all patients and to ensure financial sustainability by generating social impacts and aligning all stakeholders to improve population health and, consequently, value.
